# A Bite Reborn: Full-Mouth Rehabilitation of a Severely Worn Dentition Using a Staged Approach

**DOI:** 10.7759/cureus.107231

**Published:** 2026-04-17

**Authors:** Akhil Khadkekar, Sreeramulu Basapogu, Divya Puli, Shalini Karnam, Swapna Basimi, Marwa M Vazeer, Sadvika Juloori

**Affiliations:** 1 Prosthodontics, Government Dental College and Hospital, Hyderabad, IND

**Keywords:** full-mouth rehabilitation, occlusal harmony, provisional restorations, vertical dimension of occlusion, worn dentition

## Abstract

Severe tooth wear can result in loss of occlusal morphology, reduced vertical dimension of occlusion (VDO), compromised esthetics, and impaired function. Rehabilitation of such cases requires a systematic and reversible approach to ensure patient adaptation before definitive treatment. This report describes the rehabilitation of a 54-year-old male patient presenting with generalized attrition and reduced VDO. Following periodontal and endodontic stabilization, diagnostic mounting and wax-up were performed to establish an increase in VDO of approximately 3-4 mm. Heat-polymerized acrylic resin was used to fabricate temporary restorations (DPI Heat Cure, Dental Products of India Ltd., Mumbai, India) and maintained for six weeks to evaluate neuromuscular adaptation. Definitive restorations were fabricated using nickel-chromium alloy (Wiron 99, BEGO GmbH & Co. KG, Bremen, Germany) and veneered with feldspathic porcelain (VITA VMK Master, VITA Zahnfabrik, Bad Säckingen, Germany). Glass ionomer luting cement (GC Gold Label Type I, GC Corporation, Tokyo, Japan) was used for the final cementation. Follow-up demonstrated stable occlusion, improved esthetics, and satisfactory functional outcomes. A staged rehabilitation approach with prolonged provisionalization provides a predictable, reversible method for evaluating VDO and occlusal stability, thereby improving the long-term prognosis.

## Introduction

Tooth wear is a multifactorial condition caused by erosion, abrasion, and attrition, leading to progressive loss of tooth structure [1]. Although physiological wear occurs with aging, excessive wear can compromise function, esthetics, and occlusal stability [2]. Advanced cases often present with reduced crown height, dentin exposure, flattened occlusal surfaces, and loss of posterior support.

Reduction in the vertical dimension of occlusion (VDO) can result in altered facial proportions, decreased masticatory efficiency, and muscular fatigue [3]. Rehabilitation of such cases is clinically challenging and requires the careful evaluation of occlusion and neuromuscular adaptation.

Irreversible alteration of VDO without prior assessment may lead to temporomandibular dysfunction. Therefore, a staged and reversible approach using provisional restorations is recommended. These restorations act as a diagnostic tool, allowing the evaluation of esthetics, phonetics, and functional adaptation before definitive rehabilitation [4].

## Case presentation

**Figure 1:**
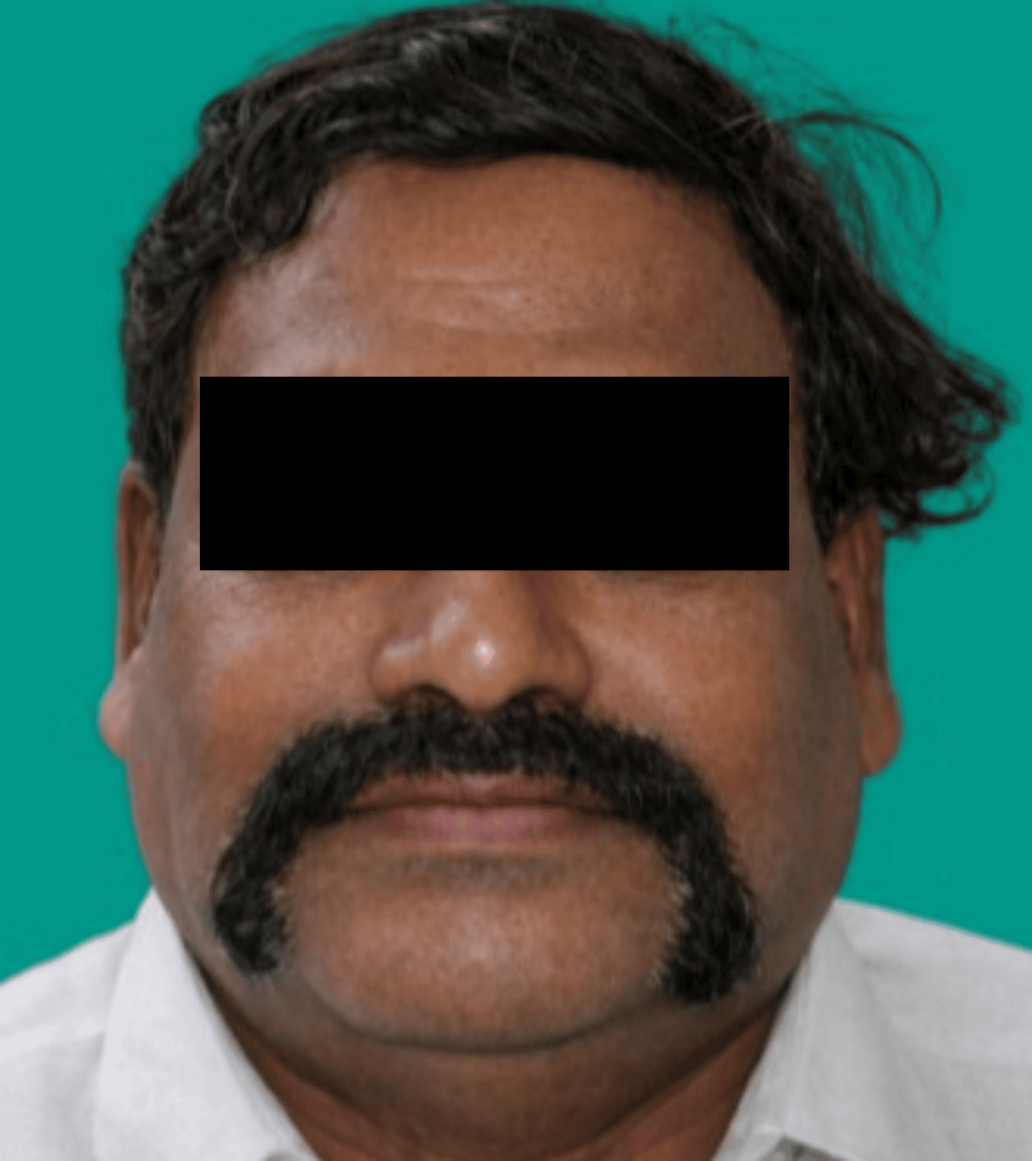
Patient on presentation

**Figure 2:**
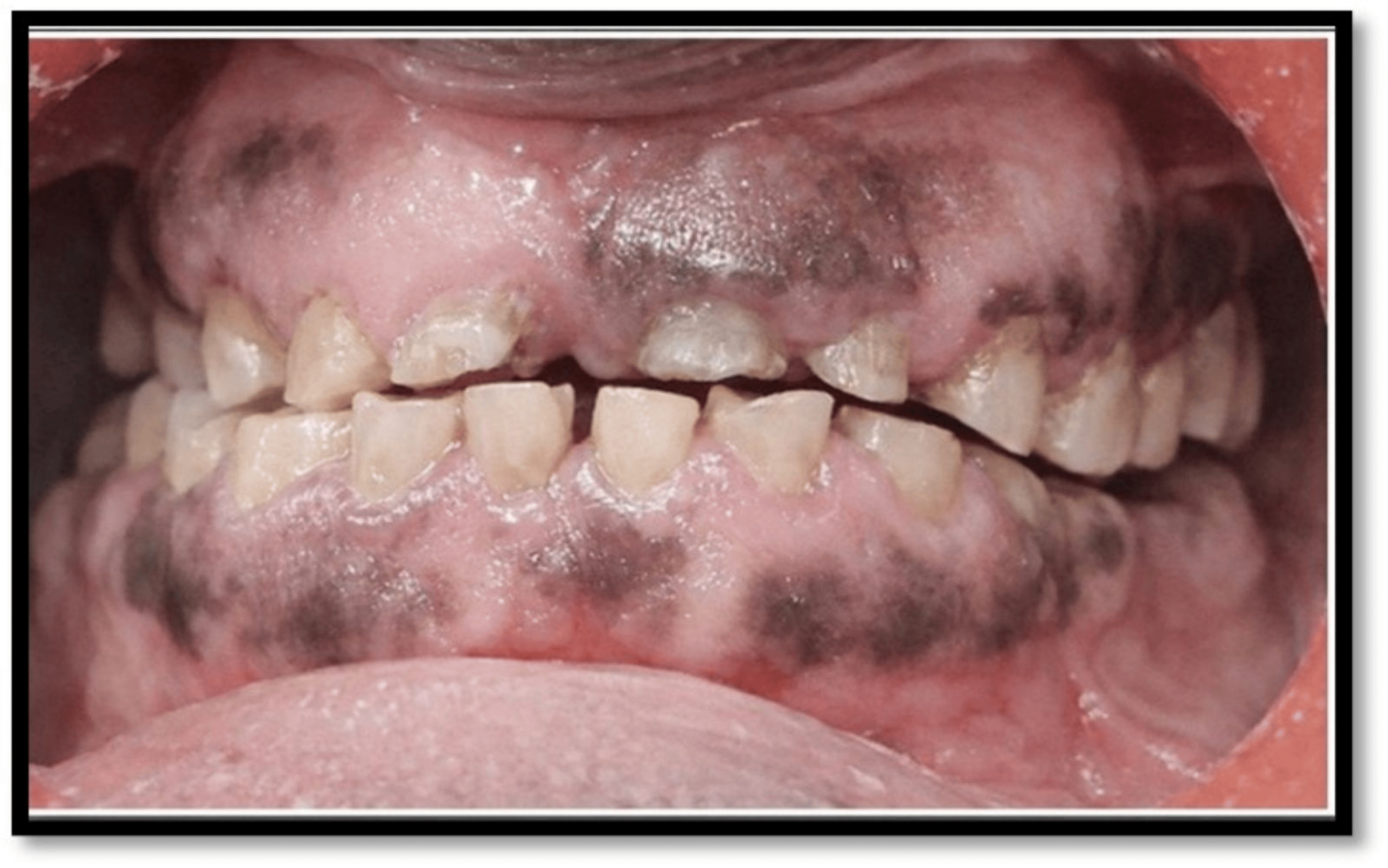
Preoperative frontal view

**Figure 3:**
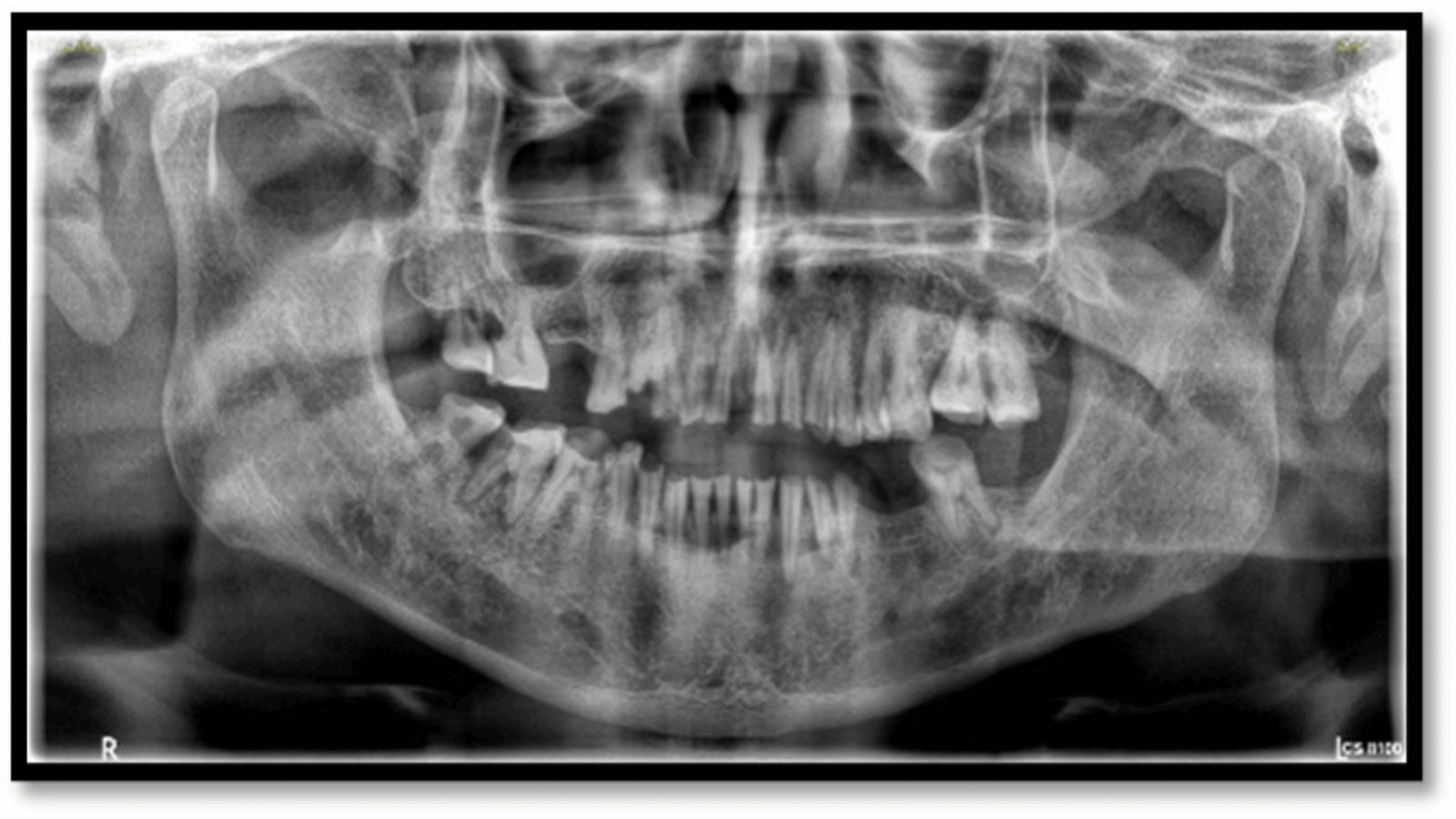
Preoperative panoramic radiograph

A 54-year-old male patient (Figure 1) presented with complaints of difficulty in mastication, generalized sensitivity, and dissatisfaction with dental appearance (Figures 2-3). His medical history was noncontributory.

Extraoral examination revealed deep nasolabial folds, increased interocclusal rest space, and reduced lower facial height. Intraoral examination showed generalized severe attrition affecting both arches, characterized by reduced clinical crown height, flattened occlusal surfaces, and dentin exposure. Posterior teeth exhibited greater wear with loss of cuspal morphology. Radiographic evaluation revealed pulpal involvement in multiple teeth. A diagnosis of generalized worn dentition with reduced VDO was established.

Clinical management

Treatment was carried out in a staged manner to ensure predictability. Occlusion was assessed using models, diagnostic impressions, and mounting. On the basis of history, clinical findings, and investigations, a diagnosis of generalized attrition with loss of vertical dimension (Turner and Missirlian Category I) was established.

**Figure 4:**
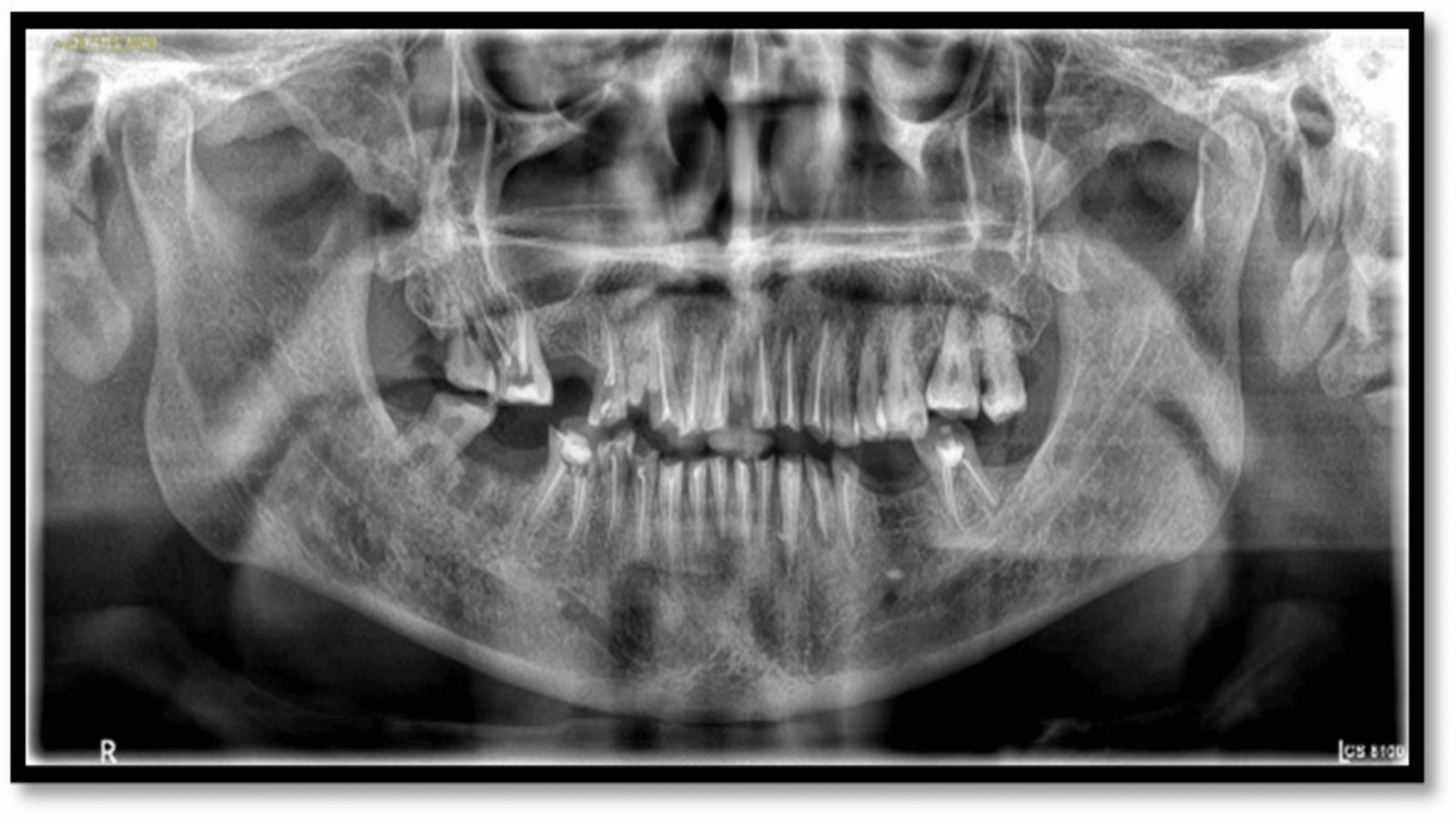
Post-endodontic panoramic radiograph

**Figure 5:**
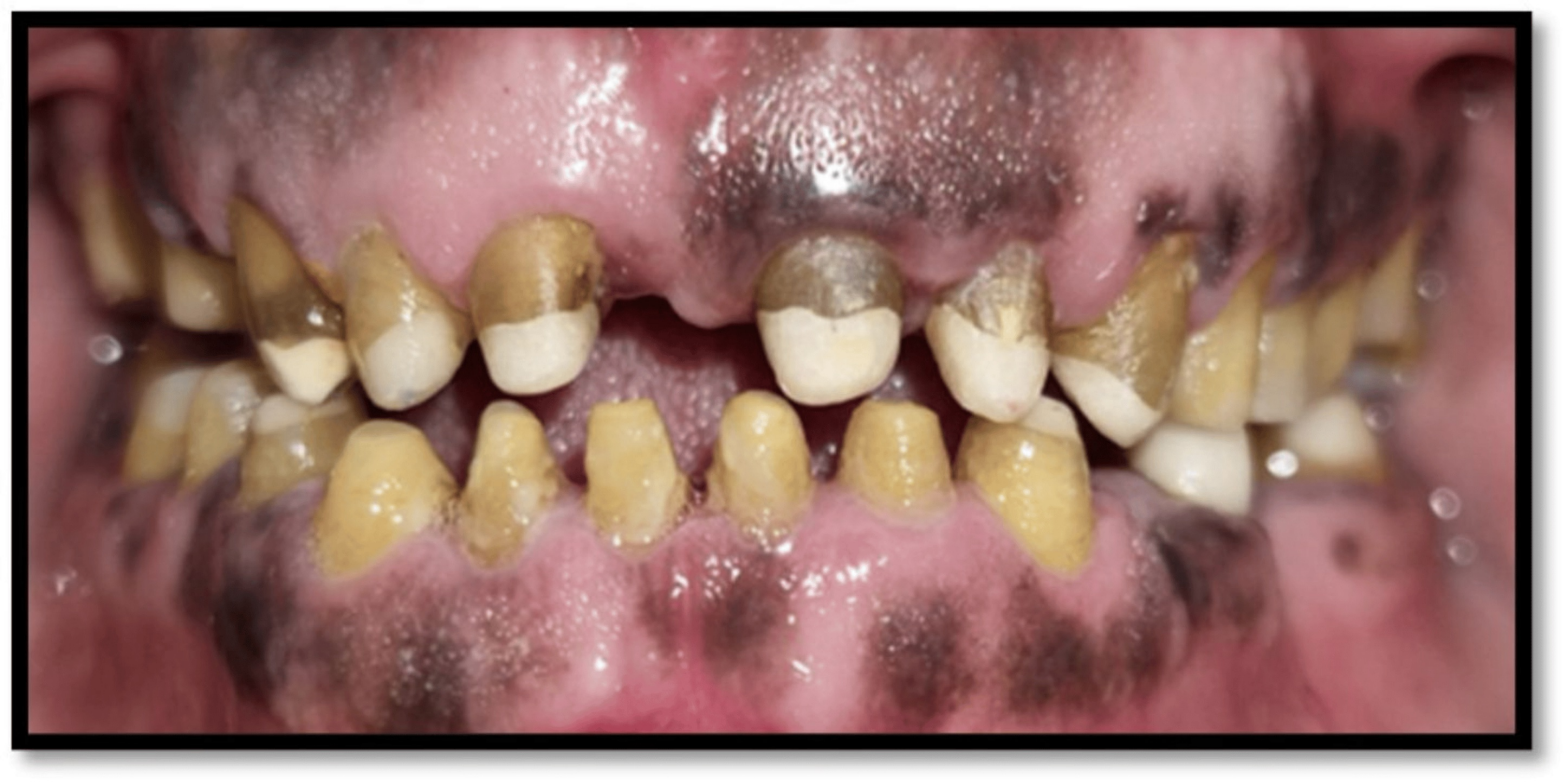
Full-mouth tooth preparation

The treatment plan was formulated with a multidisciplinary approach in various phases as follows: Phase I was advice for oral hygiene and prevention. Phase II was endodontic therapy (Figure 4) for 11, 12, 13, 14, 16, 21, 22, 23, 31, 32, 33, 34, 35, 37, 41, 42, 43, 44, 45, and 46. For Phase III, diagnostic impressions were made using irreversible hydrocolloid (Zelgan 2002, Dentsply Sirona, Gurgaon, India). A facebow transfer (Hanau Springbow, Whip Mix Corporation, Louisville, Kentucky, United States) and mounting on a semi-adjustable articulator (Hanau Wide-Vue, Whip Mix Corporation, Louisville, Kentucky, United States) were performed. Complete mock wax-up and diagnostic mounting were completed at an increased vertical dimension of 3-4 mm. Phase IV was the phase of prosthetics: Pankey-Mann-Schuyler philosophy for complete mouth rehabilitation (Figure 5).

Following the prosthetic phase, teeth preparation and prosthodontic rehabilitation were performed using the segmental arch approach and the Pankey-Mann-Schuyler philosophy as follows: mandibular anterior teeth in segment 1, maxillary anterior teeth in segment 2, mandibular posterior teeth in segment 3, and maxillary posterior teeth in segment 4.

In segment 1 of the prosthetic phase, the mandibular anterior teeth were prepared using all the guidelines for porcelain-fused-to-metal (PFM) crowns. Gingival retraction was accomplished with a gingival retraction cable, and a two-step polyvinyl siloxane (Aquasil, Dentsply Sirona, Tulsa, Oklahoma, United States) was used to create the final imprint: light body consistency and putty impression. Die preparation was done using the Pindex technique, and impressions were poured using die stone. Crowns made of autopolymerizing polymethyl methacrylate were used to finish the provisionalization of mandibular anterior teeth (DPI, India). The maxillary anterior teeth were treated in the same way. A customized anterior guide was created by selectively altering provisional crowns after the maxillary and mandibular anterior teeth were made temporary.

**Figure 6:**
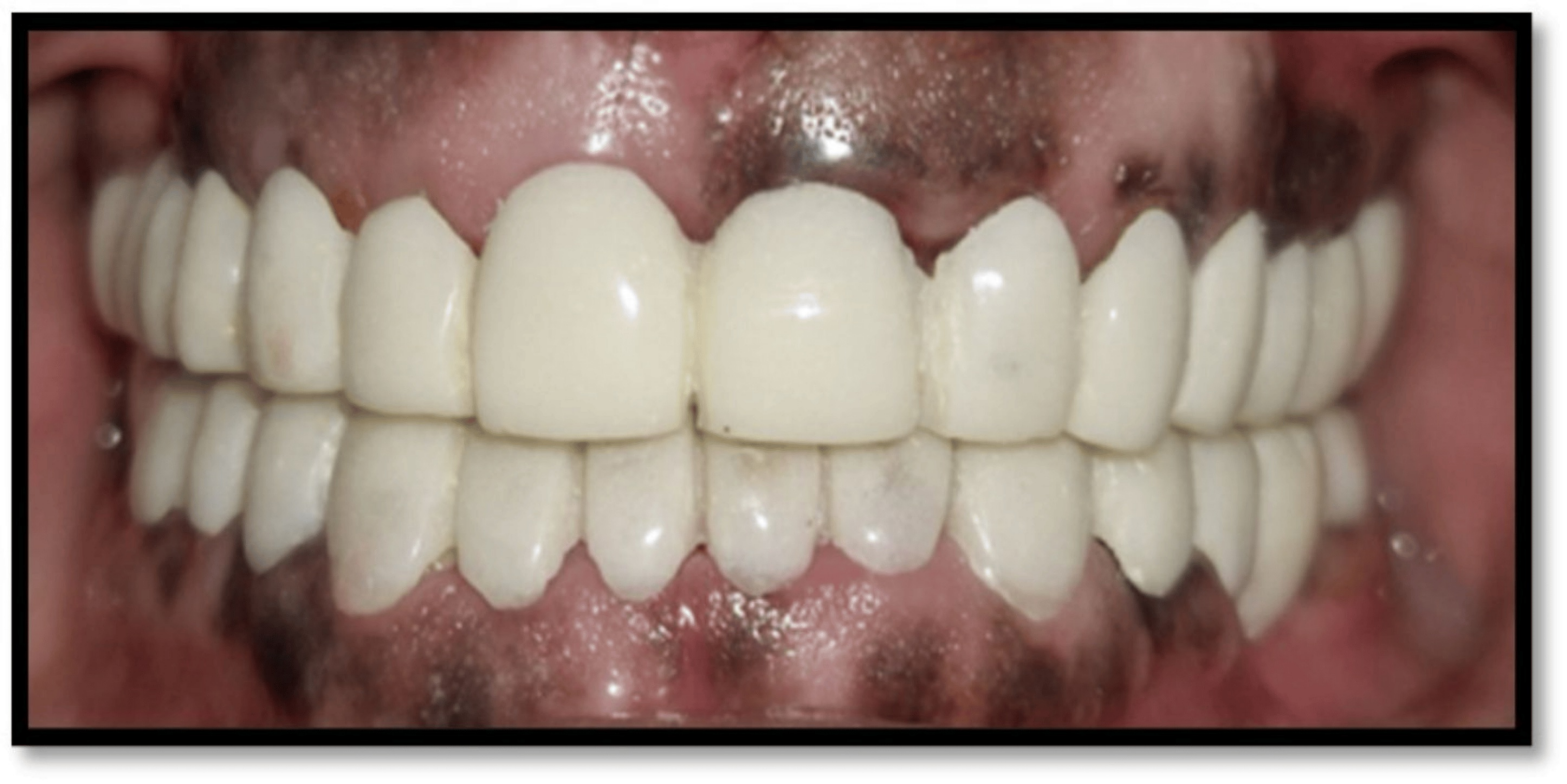
Provisional restorations

**Figure 7:**
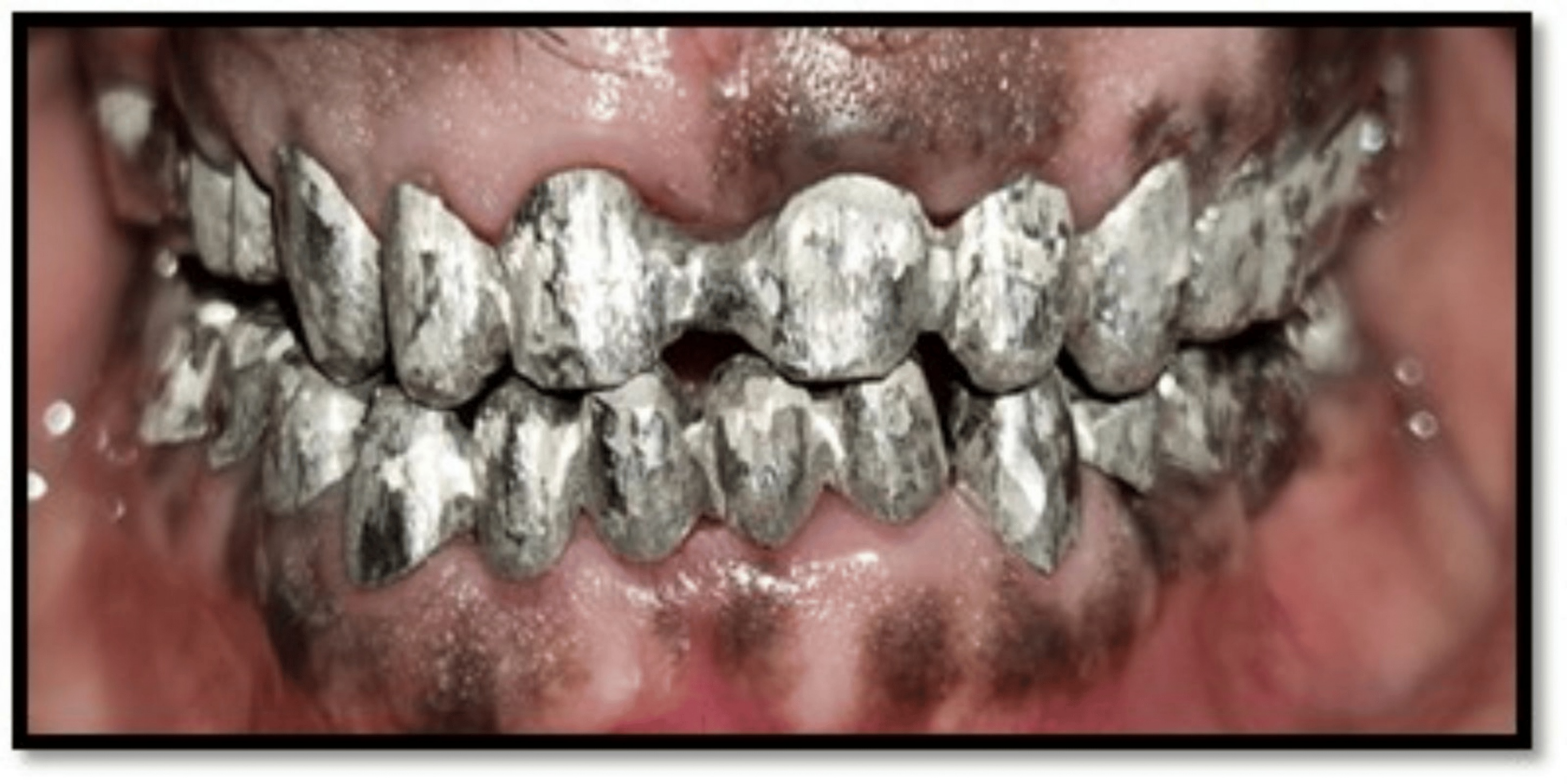
Metal framework try-in

**Figure 8:**
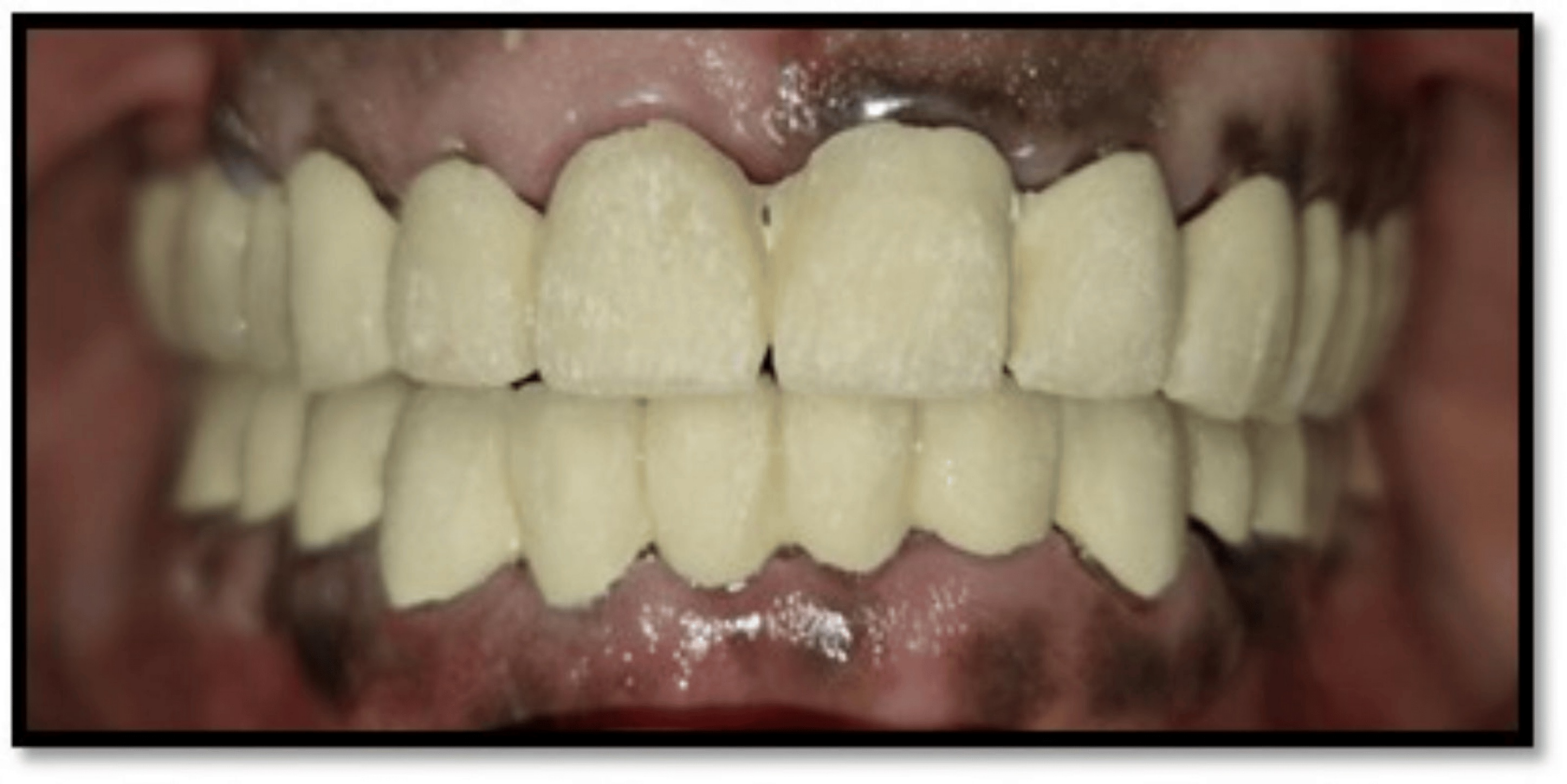
Bisque trial

Establishing a centric stop at centric relation and a smooth glide during protrusive movement are the goals of anterior guidance. At this point, a tentative repair putty index was created and replicated in definitive restorations (Figure 6). After the casting process was finished, a wax template for metal copings was created using inlay wax for the maxillary and mandibular anterior teeth. Bisque trials and metal try-ins were completed both in situ and on cast (Figures 7-8). In order to replicate the anterior guidance, a ceramic building was performed utilizing the putty index of temporary crowns. Glass ionomer cement (GC Gold Label Type I, GC Corporation, Tokyo, Japan) was used to cement PFM crowns in situ after glazing was finished.

An occlusal plane for mandibular posterior teeth was determined using Broadrick's Occlusal Plane Analyzer (Whip Mix Corporation, Louisville, Kentucky, United States), and a putty index of the estimated occlusal plane was created. All clinical procedures were followed during the preparation of teeth, final imprint, and provisionalization. The putty index guide of the estimated occlusal plane was followed for waxing up the metal copings of PFM restorations.

**Figure 9:**
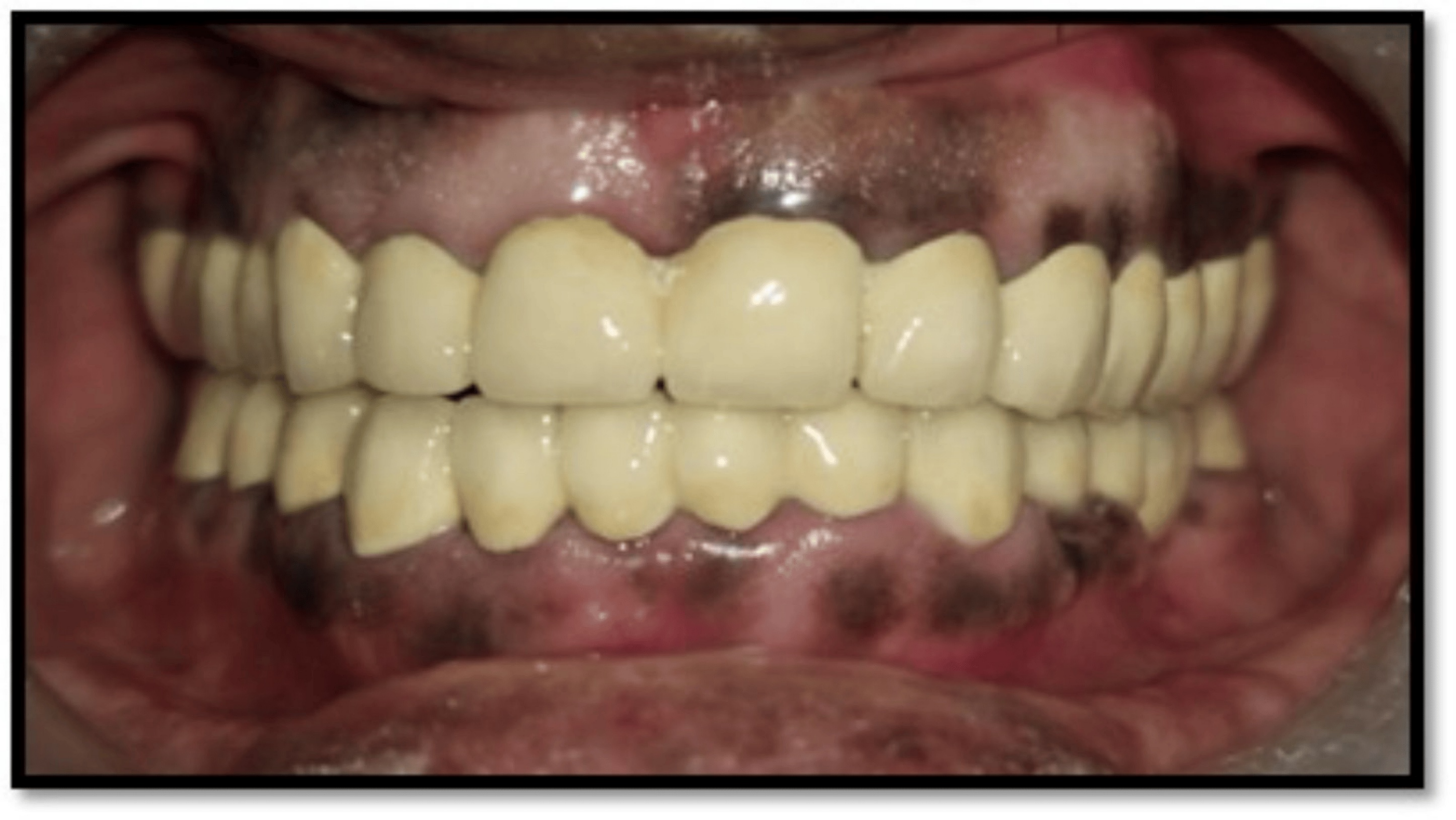
Final cementation

**Figure 10:**
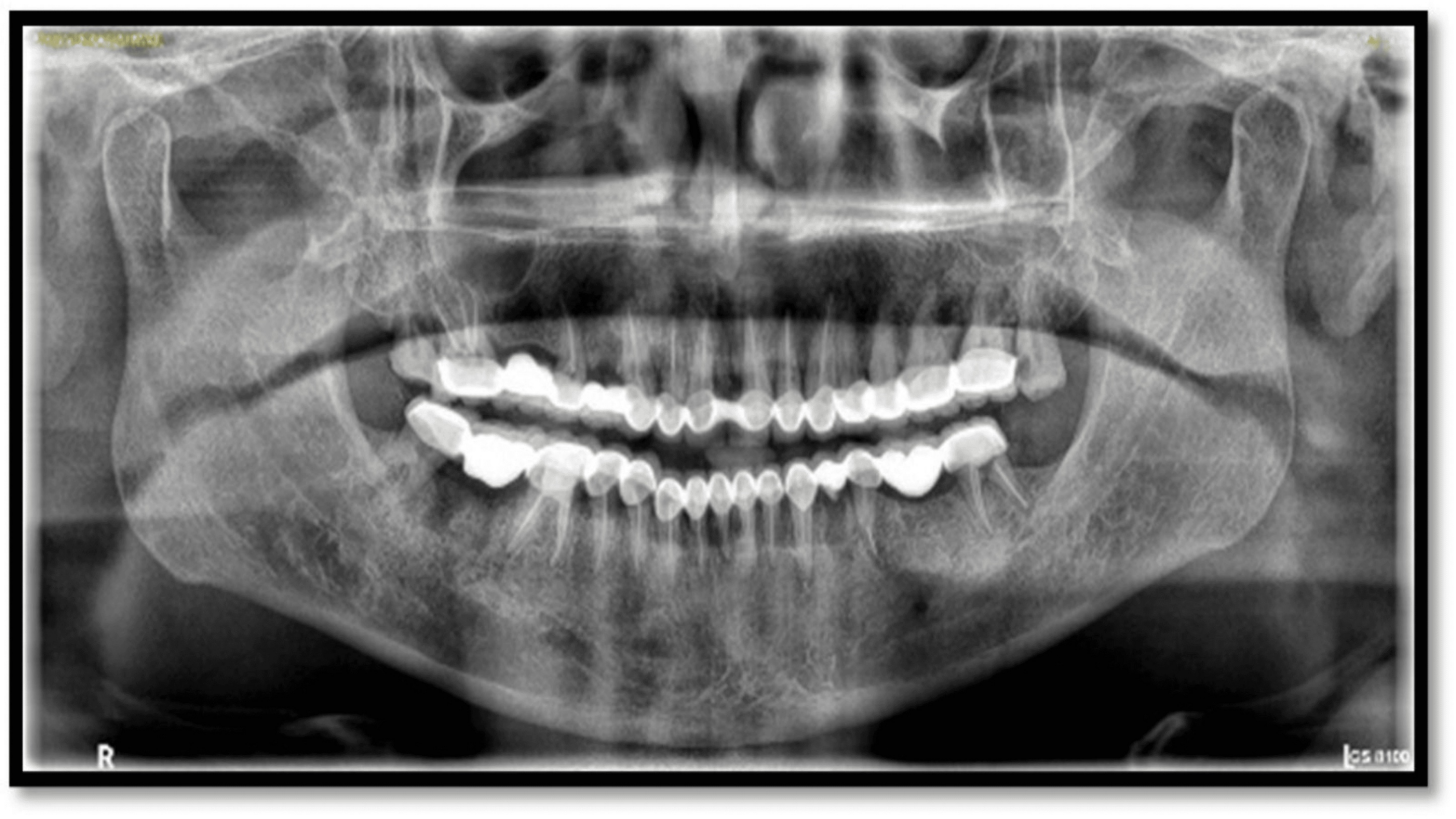
Postoperative panoramic radiograph

All mandibular posterior teeth underwent casting of the metal framework, layering of ceramic (porcelain), and final finishing. Glass ionomer cement was used to fix PFM crowns in situ after they were tested on casts. Maxillary posterior teeth rehabilitation was undertaken. Gingival retraction, tooth preparation, and final impression were completed. All laboratory procedures were followed to fabricate PFM crowns (VITA VMK Master, VITA Zahnfabrik, Bad Säckingen, Germany) and were cemented using glass ionomer cement. Occlusion was evaluated for centric and eccentric contacts with the establishment of group function (Figures 9-10). The patient's mastication, phonetics, appearance, and self-confidence were all greatly enhanced by the prosthetic rehabilitation.

Follow-up and outcomes

Follow-up at one, three, and six months revealed stable occlusion, healthy periodontal tissues, and absence of temporomandibular symptoms. The restorations demonstrated excellent marginal integrity without fracture or debonding. The patient reported improved function and esthetics, indicating a successful rehabilitation.

## Discussion

Determining a VDO that is biologically appropriate is crucial for managing worn dentition. Turner and Missirlian promoted a holistic and rigorous approach in such situations [1], whereas Branco et al. emphasized the significance of central relation stability [2].

In order to assess functional adaptation, esthetics, and patient comfort, long-term temporary restorations are used as a reversible diagnostic phase. In this instance, temporary restorations were used to gradually enhance VDO (around 3-4 mm), which was sustained over a period of six weeks. The clinical evaluation Phase IV indicated that this progressive increase ensured the lack of temporomandibular pain and allowed for sufficient neuromuscular adaptation.

The choice of materials is crucial for long-term success. While nickel-chromium alloys provide strength and durability, feldspathic porcelain gives remarkable esthetics. The long-term efficacy of full-mouth therapy depends on both material performance and patient adaptation. In this case, extended provisionalization ensured functional stability before definitive therapy, as evidenced by positive follow-up results [5].

An organized, staged, and reversible treatment method is demonstrated in this case, with a focus on extended provisionalization as a crucial stage in reaching functional stability before final recovery. Long-term provisionalization functions as a dynamic diagnostic stage that permits the ongoing evaluation of phonetics, esthetics, neuromuscular adaptability, and occlusal harmony in functional circumstances. It makes it possible to confirm the intended increase in VDO, guaranteeing patient tolerance and the lack of temporomandibular pain. Additionally, it offers a chance to improve occlusal schemes, such as creating secure posterior contacts and mutually protected occlusion, which reduces biomechanical issues and improves the predictability and durability of final restorations.

In contrast to standard procedures, this instance reduced the likelihood of temporomandibular issues by allowing enough time for neuromuscular adaptation before the insertion of permanent restorations. This example differs from standard rehabilitations in that it incorporates diagnostic wax-up, material-based planning, and documented follow-up to improve predictability [1-7].

## Conclusions

A staged approach incorporating diagnostic wax-up, prolonged provisionalization, and definitive restorations provides a predictable method for rehabilitating severely worn dentition. Verification of vertical dimension and occlusal stability prior to definitive treatment is essential for long-term success.
